# Newly qualified primary care nurses’ preparedness to make sound clinical judgements in practice

**DOI:** 10.4102/curationis.v47i1.2567

**Published:** 2024-10-03

**Authors:** Mavis Ndlela, Charlené Downing

**Affiliations:** 1 Department of Nursing, Faculty of Health Sciences, University of Johannesburg, Johannesburg, South Africa

**Keywords:** primary health care, clinical judgement, preparedness, newly qualified, quantitative research

## Abstract

**Background:**

The public health sector in South Africa provides care to more than 40 million people, with primary health care (PHC) clinics acting as most patients’ first contact with healthcare services; 80% of patients are treated by primary care nurses. Primary care nurses’ poor clinical judgment can result in poor patient care, complications and death. However, newly qualified primary care (NQPC) nurses’ preparedness for making sound clinical judgements in practice has not been evaluated in Gauteng province, South Africa.

**Objectives:**

To determine NQPC nurses’ level of preparedness in making sound clinical judgements in practice.

**Method:**

A quantitative, non-experimental, descriptive design was used. The target population was NQPC nurses. A census method was used to select all NQPC nurses in public PHC facilities in three health districts of Gauteng. The sample size comprised 77 NQPC nurses, and data were collected using a self-administered questionnaire.

**Results:**

The findings indicated that 83% of NQPC nurses were able to notice salient changes in patients’ conditions, 43% were able to correctly interpret the changes that were noticed, but only 42% prioritised the correct patient care.

**Conclusion:**

The results reflect that 50.2% of the NQPC nurses in public PHC facilities in the three districts in Gauteng were functioning at exemplary levels.

**Contribution:**

This article highlighted NQPC nurses’ level of preparedness to make sound clinical judgements within a year after qualifying.

## Introduction

Globally, health care systems are challenged by the increasing populations and the complexity of medical conditions (De Villiers 2021:2; Jessee 2021:50). South African health system has its own challenges of the shortage of medical and nursing staff against a rise in the quadruple burden of disease, that is, high rates of HIV, AIDS and tuberculosis (TB); maternal and child mortality; hypertension and cardiovascular diseases, diabetes, injury and trauma (Kordom, Daniels & Chipps 2023:3). Along with strategies to establish universal health coverage for all, there is a constitutional obligation to offer quality care in all health facilities, including primary health care (PHC) facilities (Maphumulo & Bhengu 2019:6). The PHC facilities are most patients’ first contact with the health system (Bresick et al. 2019:109), and in South Africa, PHC is provided through a nurse-based, doctor-supported infrastructure (Mckenzie et al. 2018:6). Nurses working in these facilities should be skilled and able to make sound clinical judgements. When indicators of condition deterioration are unrecognised or ineffectively managed, they affect patients’ health outcomes and result in complications (Dresser 2019:1). This calls for all newly qualified primary care (NQPC) nurses entering practice to be ready to make sound clinical judgements.

**FIGURE 1:**
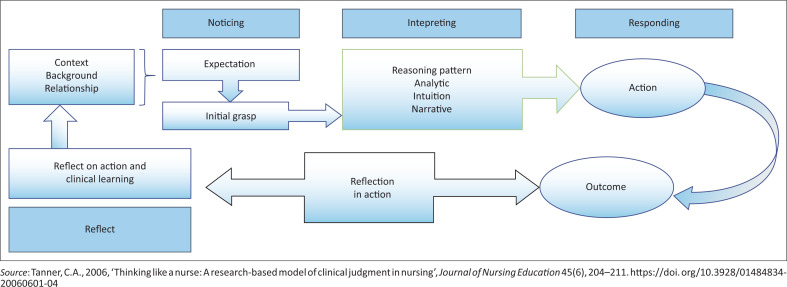
Clinical judgement model.

Tanner’s (2006:204) clinical judgement model defines clinical judgement as the ability to notice salient changes in a patient’s condition, interpret the changes and respond to the changes while reflecting on the care to be given (Tanner 2006:205). The clinical judgement model comprises the four dimensions, (1) noticing, (2) interpreting, (3) responding and (4) reflecting in action and on action (Figure 1). The newly qualified PHC nurses demonstrate their skill by reflecting *in* action when noticing, interpreting and deciding on the best management option for the patient based on previous clinical experience of managing similar cases (Mohamed & Albeladi 2020:2; Tanner 2006:204). They also reflect *on* the actual action after the care was rendered. It is a critical practice in the development of clinical knowledge and improvement in clinical reasoning.

Clinical judgement requires flexibility and the ability to notice salient aspects of a clinical presentation, interpret the changes correctly and respond in a precise manner (Tanner 2006:207). It is guided by the nurses’ theoretical knowledge and practical skills to prioritise patients’ needs (Lawrence et al. 2018:22; Tanner 2006:205; Van Graan, Williams & Koen 2016:35). Clinical judgement is built on knowledge, clinical experience, critical thinking, reasoning, intuition and evidence-based practice abilities (Kinyon, D’Alton, Poston & Navarrete 2021:600).

In South Africa, the PHC facilities comprise 77% nurses and 11% medical doctors (Bresick et al. 2019:110). In South Africa, primary care nurses are nurse specialists in advanced clinical practice and have completed a basic nursing qualification and registered with the South African Nursing Council (SANC) Regulation R.425 as nurse (General, Psychiatric and Community) and midwife and completed a 1-year post-basic diploma in clinical health assessment, treatment and care and are registered with the SANC Regulation 48 (R.48) as PHC nurse specialists (Kordom et al. 2023:2). At the time of the study, there were no NQPC nurses in the new programme postgraduate diploma in primary care (R.635). The training of PHC nurses equips them with the knowledge and skills to assess, diagnose and treat patients of all ages presenting with minor and complex medical and surgical conditions, manage all emergencies in PHC facilities and, where necessary, refer them appropriately to the next level of care (Marsh et al. 2016:33; Mofolo, Heunis & Kigozi 2019:2). This calls for all NQPC nurses entering practice to be ready to ‘hit the floor running’ by making sound clinical judgements (Kaya, Senyuva & Bodur 2018:26; Lawrence et al. 2018:22; Maphumulo & Bhengu 2019:8; Tuomikoski et al. 2018:79).

Sound clinical judgement is a complex process dependent on cumulative knowledge and experience to accurately notice salient clinical symptoms and appropriately interpret and execute a safe management plan (Lawrence et al. 2018:23; Tanner 2006:205; Van Graan et al. 2016:34). However, increases in patient acuity and the complexity of health care system challenge PHC nurses’ competencies (Duff 2019:145). The NQPC nurses are not supported in clinical facilities soon after their training, and this has a negative impact on patient outcomes (Madalane 2019:5).Moreover, the quadruple burden of disease and the shortage of health personnel further challenge newly qualified nurses entering practice into making clinical decisions (Madalane 2019:3; Maphumulo & Bhengu 2019:49). There are also concerns that nurses entering practice are often perceived as not meeting expectations for providing safe nursing practice in a complex environment (Jacobs et al. 2018:747; Rusch et al. 2019:37).

Newly qualified primary care nurses’ failure to make sound clinical judgements can cause patients to suffer complications, unnecessary pain, emotional trauma and loss of income, with ensuing litigations depleting funds that could be used for medicine, equipment and staff salaries (Edeling & Claasen 2018:43; South African Law Reform Commission 2017:3). Studies have been conducted in the preparedness of newly qualified nurses in other disciplines, and yet there is no study conducted in the preparedness of NQPC nurses. Therefore, this study aims to determine the NQPC nurses’ level of preparedness to make sound clinical judgements in practice. In making sound clinical judgements, the NQPC nurses become adaptable to various challenging clinical situations and can make quick, life-saving decisions that ensure quality patient care.

## Research methods and design

### Study design

A non-experimental quantitative, descriptive research design was used to identify and measure NQPC nurses’ preparedness to make sound clinical judgements in practice within a year after qualifying as advanced practice nurses (Gray & Grove 2021:234, 247; Melnyk & Fineout-Overholt 2019:622; Polit & Beck 2021:162). The design was used as it is non-experimental requiring no manipulation of the independent variable in the study while measuring the level of preparedness of NQPC nurses.

### Study setting

The study was conducted in public PHC facilities in Ekurhuleni, Johannesburg and the Tshwane Metropolitan Municipalities. These metropolitan municipalities are three out of five metropolitan municipalities in Gauteng, South Africa. A total of 64 PHC facilities were used, of which 38 were from Ekurhuleni, 15 from Johannesburg and 11 from Tshwane Metropolitan Municipality. Gauteng was selected as it had the last group of the R.48 students in training following the SANC Circular 5/2019, which granted the special concession for a final intake of students in the legacy qualification before December 2019. The students in the new primary care qualification R.635 were still on training during data collection. This selection was further made necessary by the national lockdown because of coronavirus disease 2019 (COVID-19), as Gauteng became the most accessible province for the authors. The study focused on public PHC facilities offering a full PHC package of services, with NQPC nurses providing care. The comprehensive PHC package includes prevention, promotion, curative and rehabilitative services; ward-based outreach health services; immunisations; integrated management of childhood infections (IMCI); sexual reproductive health services; antenatal care; screening for cervical and prostate cancer; TB diagnosis and treatment; patient or health provider-initiated counselling and testing for HIV; nurse initiation of antiretroviral treatment; diagnosis and treatment of minor ailments; diagnosis and management of diseases of the lifestyle; school health services and emergency management at the primary level (Department of Health 1997).

### Population and sampling

The study’s population was primary care nurses working at public PHC clinics in South Africa. The target population was all NQPC nurses working in public PHC facilities in Tshwane, Johannesburg and Ekurhuleni health districts in Gauteng province. A purposive sampling method was used to select all NQPC nurses willing to participate as they were most likely to provide the appropriate information for the study (Gray & Grove 2021:411; Polit & Beck 2021:261). The purposive sampling method was used to purposefully select study respondents who could provide the best information to achieve the study’s objectives based on their characteristics, experience and knowledge (Campbell et al. 2020:653).

### Inclusion criteria

- All NQPC nurses who qualified within the last 12 months.
- All NQPC nurses working in public PHC facilities in the Johannesburg, Ekurhuleni and Tshwane Metropolitan Municipalities.

### Exclusion criteria

- Primary care nurses qualified for more than 12 months were excluded.
- Newly qualified primary care nurses not working in primary care facilities as they were not exposed to making PHC decisions.
- Newly qualified primary care nurses employed in private PHC facilities were excluded.

### Pilot study

A pilot study was conducted on 4% (*n* = 7) of the sample (*N* = 166). The results were used to test the feasibility of the data collection instrument. Lessons learned during the pilot study were used to make corrections to the questionnaire for use in the final study. The results from the pilot study were not used in the study findings.

### Instrument

Data were collected using the Lasater Clinical Judgement Rubric (LCJR) developed by Dr Kathy Lasater (Shin, Park & Shim 2015:69; Vreugdenhil & Spek 2018:44; Yang et al. 2019:6). No changes were made on the LCJR. The rubric contained the four phases of Tanner’s clinical judgement model, namely noticing, interpreting, responding and reflecting. Each of these four phases was further described by 11 dimensions that clarified each phase by providing a detailed description of expected behaviour levels (Georg et al. 2019:15).

The questionnaire had two sections: section A focused on respondents’ demographic information, such as ethnicity, for generalising the findings to all races, age and years of experience after obtaining their first professional qualification as older and seasoned nurses could be more qualified to assess intricate circumstances, identify minute modifications in patients’ conditions and foresee possible issues. Section B comprised two unfolding case studies ranging from simple to complex conditions. The case studies were extracted from the Nursing.com website, Primary Care Case Studies 101 and Teaching Clinical Judgement Through Cases. The extracted cases aligned with medical conditions prevalent in PHC facilities in South Africa. Each case scenario’s pharmacological management was adapted to comply with the 2018 Primary Health Care Standard Treatment Guidelines and Essential Medicine List of South Africa and the management protocols or treatment guidelines relevant to PHC clinics in South Africa (see Appendix 1 for an example of a case study).

Each case study contained 11 questions with a rating of 1 to 4. The questions were multiple choice and open ended to determine NQPC nurses’ ability to notice cues in a patient’s condition, interpret the cues based on knowledge, intuition, experience and higher-order thinking and respond through actions based on higher-order thinking skills and experience. Their ability to assess the outcomes of the treatment modality, put actions into practice and reflect on actions and what could have been done, and with what effect, were also considered.

An ordinal scoring tool was used to provide a detailed description of the expected performance (Marsh et al. 2016:33) using the four processes of noticing, interpreting, responding and reflecting. These processes were operationalised in 11 items, which meant 11–44 points could be scored per case. Each section had an unfolding case study. One was an IMCI case, and the second was a complex case of diabetes with a comorbidity. The questions addressed respondents’ knowledge, skills, clinical reasoning, critical thinking and decision making.

The overall scores ranged from 22 to 88, with the different levels indicating respondents’ levels of preparedness in making sound clinical judgements. Total scores of 22 indicated clinical reasoning skills at the beginner level, 23–44 the developing level, 45–66 the accomplished level and 67–88 the exemplary level. Higher scores thus indicated heightened levels of clinical judgement.

There were no changes made to the data collection tool; the LCJR was validated in the United States of America (USA) (Adamson et al. 2012:70; Knipe 2013; Sideras 2007:5; Strickland, Cheshire & March 2017:86; Victor-Chmil 2013:35) and translated and validated in South Korea (Shin et al. 2015:70). Validation was conducted in academic simulation settings. In Sweden, the Cronbach’s alpha was 0.86; in South Korea, it was 0.90 and in the USA, it was 0.97. In Sweden, the intraclass coefficient was 0.86, and in the USA, it was 0.89. In this study, the LCJR was reliable, with a Cronbach’s alpha of 0.89.

### Data collection procedure

The complexity of the COVID-19 regulations of social distancing and restrictions of non-medical-related visits to clinical facilities required the authors to employ diverse approaches to optimise data collection. The first author thus used an online data collection method (Google Forms) during the phase when access to clinical facilities was not permitted. The information letter with the contact details of the researcher and a consent form was forwarded to prospective respondents’ email addresses. Prospective respondents who required more information were able to contact the first author. On completing the consent form, the questionnaire was then accessible to prospective respondents with email addresses. The email addresses of the NQPCN were on the student database of the public nursing education institutions and the training coordinators of the various Metropolitan municipalities were also in possession of the details of NQPCN from the respective Metropolitan Health districts. Upon obtaining permission to collect data from the Department of Health, the gatekeepers of the institution (clinic managers and training coordinators in certain municipalities) forwarded the information to the prospective respondents who then responded from the email forwarded by gatekeepers. After COVID-19 restrictions were relaxed and access to clinical facilities was permitted, the first author visited facilities in the Ekurhuleni, Johannesburg and Tshwane Municipalities to provide an information session and request participation from prospective respondents who were not accessible through the emails. Not all NQPC nurses were available in the facilities visited as some have terminated their service and could not be accessed. Consent forms were signed before the NQPC nurses completed the questionnaire. Data were collected from October 2021 to December 2021, and in this period, there were no NQPC nurses from the new programme, R.635.

The sample frame was 166 NQPC nurses; 121 questionnaires were forwarded to prospective respondents who were accessible to the researcher through email and those without an email but were within the physical reach of the researcher and within the districts where data were collected. Ninety-six online forms were forwarded to prospective respondents’ email addresses, 29 were completed and 67 returned as undelivered with invalid email addresses. Seventy-three hard copies were hand delivered, and 48 responses were returned completed. A total of 77 completed responses were received, yielding the attrition rate of 28.7%. The attrition rate exceeded the expected percentage (Polit & Beck 2021:261).

### Data analysis

Preparing data collected included cleaning, structuring and reformatting data to eliminate missing values, inaccuracies, anomalies or other errors. Data were analysed using Statistical Package for Social Sciences (SPSS) software, version 27, with the assistance of the supervisor and Statcon services from the University of Johannesburg. The statistical methods of analysis included descriptive, inferential and correlational analyses.

Descriptive statistics were used to describe the data’s distribution using the measures of central tendency (mean, median, percentiles, standard deviations and ranges). Inferential statistics are used to predict the parameters of a population based on the sample of available data (McQuoid-Mason 2018:490). The study used the analysis for variance (ANOVA) test, and correlation analysis was conducted to investigate whether variables of clinical judgement were related if they reacted differently or similarly and if they measured the same dimension (Creswell & Creswell 2018:164). A Spearman’s (rho) non-parametric test was conducted to examine the degree and direction of the association between clinical judgement variables (Gray & Grove 2021:168; Melnyk & Fineout-Overholt 2019:642).

### Validity and reliability

The study used the data collection instrument, LCJR, which has been tested for its ability to consistently measure the concept being researched. The 11 dimensions of the LCJR exhibited strong internal consistency and produced findings of 0.86–0.90, with the four phases producing Cronbach’s alpha (0.83, 0.91, 0.92, 0.93), demonstrating internal consistency between the two faculty raters and supporting reliability (Manetti 2015:49). The LCJR covered the full domain associated with the variable or construct of clinical judgement.

### Ethical considerations

Permission to conduct the study was granted by the University of Johannesburg’s Faculty of Health Sciences Research Ethics Committee (REC-625-2020), Higher Degrees Committee (HDC-01-50-2020), Johannesburg Health District Research Committee (GP 202106 064), Johannesburg Health District, Ekurhuleni Health District, Tshwane Health District, Thelle Mogoerane Regional Hospital and Hellen Joseph Hospital before commencing data collection. All respondents completed and signed an informed consent form before data collection commenced. Respondents using the online questionnaire were forwarded an information letter and a consent form. After reading the information letter, those wishing to participate would click to access the consent form, which they completed and submitted before they could access the questionnaire. The authors adhered to all principles of justice, autonomy, beneficence and non-maleficence before, during and after data collection (McQuoid-Mason 2018:490).

## Results

A total of *n* = 77 respondents completed the questionnaire and yielded a response rate of 63.6%. The response rate of 60% is relatively good and can yield reliable data (Fowler 2013:17; Polit & Beck 2021:261). Table 1 describes all the respondents’ demographic characteristics. The respondents’ mean age was 31 years. Most (48%; *n* = 37) respondents had more than 3 years of experience as professional nurses. Their employment history in PHC facilities was included since the context and culture in which nurses practise influence their development of clinical nursing expertise (Fawaz & Hamdan-Mansour 2016:38). In this study, 57% of respondents had worked in PHC facilities for 3 years or longer. In this study, 57% of respondents had worked in PHC facilities for 3 years or longer (Table 1).

**TABLE 1:** Respondents’ demographic details.

Demographic variable	*n*	%
**Age (years)**		
25–30	26	33.8
31–40	22	28.5
41–50	14	18.2
51–60	14	18.2
61 or older	1	1.3
**Gender**		
Female	66	85.7
Male	10	13.0
Prefer not to answer	1	1.3
**Ethnicity**		
Black people	66	85.7
Coloured people	7	9.1
Asian people	4	5.2
**Highest qualification**		
Post basic diploma	67	87.0
Bachelor’s degree (postgraduate)	10	13.0
**Professional experience (years)**		
Less than 1	1	1.3
1–2	11	14.3
2–3	28	36.4
3 or more	37	48.0
**Experience in PHC (years)**		
Less than 1	6	7.8
1–2	13	16.9
2–3	14	18.2
3 or more	44	57.1
**Experience in trauma or ICU (years)**		
Less than 1	54	70.1
1–2	9	11.7
2–3	7	9.1
3 or more	7	9.1
**Experience in ICU (years)**		
Less than 1	65	84.4
1–2	9	11.7
3 or more	3	3.9

*Source:* Adapted from Ndlela, M., 2022, ‘Preparedness for sound clinical judgement in practice: Newly Qualified Primary Health Care Nurses’, Master’s thesis, Dept. of Nursing, University of Johannesburg, viewed 20 August 2024, from https://hdl.handle.net/10210/503861

PHC, primary health care; ICU, intensive care unit.

### Case study 1

The case was a simple and common paediatric condition of gastroenteritis with dehydration. The NQPC nurses had to diagnose the case and decide on the type and volume of fluids needed to rehydrate the patient and the nursing care required while awaiting transportation to the hospital. The results of Case study 1 are depicted in Table 2.

**TABLE 2:** Findings of Case study 1 and Case study 2.

Variable	Unit	Case study 1				Case study 2			
		Beginning	Developing	Accomplished	Exemplary	Beginning	Developing	Accomplished	Exemplary
Focused observation	*n*	4	3	1	69	-	12	46	19
	%	5.2	3.9	1.3	89.6	-	15.6	59.7	24.7
Recognising deviations from	*n*	-	5	4	68	-	-	2	75
	%	-	6.5	5.2	88.3	-	-	2.6	97.4
Information seeking	*n*	2	7	12	56	6	4	4	63
	%	2.6	9.1	15.6	72.7	7.8	5.2	5.2	81.8
Prioritising data	*n*	14	8	28	27	40	24	12	1
	%	18.2	10.4	36.4	35.1	51.9	31.2	15.6	1.3
Making sense of data	*n*	24	-	13	40	7	24	24	22
	%	31.2	-	16.9	51.9	9.1	31.2	31.2	28.6
Calm, confident manner	*n*	17	16	19	25	9	5	35	28
	%	22.1	20.8	24.7	32.5	11.7	6.5	45.5	36.4
Clear communication	*n*	14	20	24	18	15	13	25	24
	%	18.4	26.3	31.6	23.7	19.4	16.8	32.4	31.1
Well-planned intervention	*n*	7	14	13	42	12	33	22	10
	%	9.2	18.4	17.1	55.3	15.6	42.9	28.6	13.0
Being skilful	*n*	10	13	23	31	33	33	8	3
	%	13.0	16.9	29.9	40.3	42.9	42.9	10.4	3.9
Evaluation, self-analysis	*n*	-	2	5	70	19	26	21	11
	%	-	2.6	6.5	90.9	24.7	33.8	27.3	14.3
Commitment to improvement	*n*	2	25	29	21	39	19	15	4
	%	2.6	32.5	37.7	27.3	50.6	24.7	19.5	5.2

*Source:* Adapted from Ndlela, M., 2022, ‘Preparedness for sound clinical judgement in practice: Newly Qualified Primary Health Care Nurses’, Master’s thesis, Dept. of Nursing, University of Johannesburg, viewed 20 August 2024, from https://hdl.handle.net/10210/503861

**Noticing:** Noticing dimensions includes focused observation, noticing deviations and seeking correct information. Focussed observation was evident among *n* = 69 (89.6%) respondents at the exemplary level and *n* = 7 (9.1%) at the developing and beginner level. Moreover, *n* = 68 (88.3%) respondents were able to recognise deviations through the presenting signs and functioned at an exemplary level. In seeking the correct information, *n* = 56 (72.7%) of respondents were at an exemplary level, while *n* = 9 (11.7%) functioned at the beginner and developing level.

**Interpreting:** In interpreting, only *n* = 27 (35.1%) respondents functioned at an exemplary level and were able to prioritise data correctly, while *n* = 28 (36.4%) were at the accomplished level and *n* = 14 (18.2%) at the beginner level. In making sense of the data, *n* = 40 (51.9%) respondents were at an accomplished level, while *n* = 7 (9.1%) were at an exemplary level. Twenty-four (31.2%) respondents were at beginner level and unable to make sense of data.

**Responding:** In responding, *n* = 25 (32.5%) respondents were calm and acted sequentially at the exemplary level, while *n* = 17 (22.1%) were at the beginner level and *n* = 16 (20.8%) at the developing level. In executing a well-planned intervention, *n* = 43 (55.3%) were functioning at an exemplary level, while *n* = 13 (17.1%) were functioning at an accomplished level, and *n* = 7 (9.2%) were at a beginner level. In being skilful, *n* = 31 (40.3%) were functioning at an exemplary level, *n* = 23 (29.9%) were at an accomplished level and *n* = 10 (13%) were functioning at a beginner level.

**Reflecting:** In reflecting on action, *n* = 70 (90.9%) respondents were functioning at an exemplary level by reflecting that they could have done better, while *n* = 2 (2.6%) were at a developing level, not reflecting on what they could have done better. In committing to improvement, *n* = 21 (27.3%) respondents were at exemplary level and committed to improving, while *n* = 2 (2.6%) were at beginner level and did not identify areas for improvement.

### Case study 2

Case study 2 was a complex but common case of hypoglycaemia with dehydration.

**Noticing:** In noticing, *n* = 19 (24.7%) respondents were at exemplary level and reflected correct focused observation. In recognising deviations from expected patterns, *n* = 75 (97.4%) were at an exemplary level, though they could not link the deviations to the impending medical condition. In seeking the correct information, *n* = 63 (81.8%) were at an exemplary level as they were able to seek the correct information.

**Interpreting:** In interpreting, *n* = 1 (1.3%) respondent was at exemplary level as they were able to correctly prioritise the data, with *n* = 64 (83.1%) functioning at the beginner level and unable to prioritise data. In making sense of data, *n* = 31 (40.3%) respondents functioned at beginner level as they were unable to make sense of data, while *n* = 22 (28.6%) were at exemplary level as they were able to make sense of the data.

**Responding:** The findings highlighted that *n* = 28 (36.4%) respondents were at exemplary level and able to consider all facts when responding. In giving clear communication and directions, *n* = 24 (31.1%) respondents were at an exemplary level as they were able to give clear communication and directions in managing the case. In providing a well-planned intervention, *n* = 10 (13.0%) respondents were at exemplary level and flexible in executing well-planned interventions to address unfolding changes. In being skilful, *n* = 3 (3.9%) respondents were skilful in their nursing interventions at an exemplary level, leaving *n* = 66 (86.8%) at beginner and developing levels.

**Reflecting:** In reflecting on action, *n* = 11 (14.3%) respondents were exemplary in giving a true reflection of gaps in their own practice, while *n* = 66 (85.7%) were at beginner and developing levels as they could not find any skills gap in their actions. In responding to a need to improve, *n* = 39 (50.6%) respondents were at the development level as they identified the need to improve.

### Descriptive analysis

The descriptive results indicated respondents’ varied responses to the case studies. As depicted in the findings, the mean scores on ‘noticing’ in the case studies varied between 3.7 and 3.1, respectively. The respondents noticed the least in Case study 2.

In recognising deviations from the expected patterns, the mean scores ranged between 3.8 and 3.9. The respondents were best able to identify deviations from expected patterns in Case study 2 and recognised the most minor deviations in Case study 1. In making sense of the data, the mean scores were 2.8 and 2.7, respectively. The respondents were better able to make sense of data in Case study 1 and made the slightest sense of data in Case study 2.

The mean scores for well-planned intervention or flexibility were 3.1 and 2.3, respectively. The respondents were able to have a well-planned intervention and were more flexible in Case study 1 and least flexible in Case study 2. On being skilful, the mean scores were 2.9 and 1.7, respectively, with the highest mean scores in Case study 1 and the lowest in Case study 2.

In Case study 1, the estimated marginal mean was 35.078, with a lower bound of 34.232 and an upper bound of 35.924. The standard error was 0.425, and the confidence interval was 95%.

The consolidated score for each case was different from each question rating. In Case study 1, *n* = 9 were at the developing stage, while *n* = 24 were at the accomplished level and *n* = 44 respondents were at the exemplary level. In case 2, *n* = 3 respondents were at the beginner level, *n* = 61 were at the developing level and *n* = 13 were at the accomplished level. None of the respondents were at an exemplary level.

### Correlational analysis

A Spearman’s (rho) non-parametric test was conducted to examine the degree and direction of the link between the clinical judgement variables (Gray & Grove 2021:561). A statistician conducted the correlation on the 22 items of clinical judgement. Some items had low scores, indicating relationships between the items and the lack of critical thinking during patient assessment and management (Table 3).

**TABLE 3:** Correlation analysis matrix.

Variable	Focused observation	Recognising deviations from expected patterns	Information seeking	Prioritising data	Making sense of data	Calm confident manner	Clear communication	Well-planned intervention, flexibility	Being skilful	Evaluation, self-analysis	Commitment to improvement
Focused observation	1.000	0.448	0.030	0.166	−0.009	0.006	−0.134	−0.236	0.335	0.169	0.234
Recognising deviations	-	1.000	0.172	0.021	−0.038	−0.076	0.028	−0.128	0.281	0.029	0.270
Information seeking	-	-	1.000	−0.027	0.130	0.188	−0.033	0.173	−0.131	−0.071	−0.016
Prioritising data	-	-	-	1.000	0.133	0.112	0.061	−0.013	0.111	0.059	−0.012
Making sense of data	-	-	-	-	1.000	0.099	0.104	0.031	−0.316	0.102	−0.010
Calm and confident	-	-	-	-	-	1.000	0.119	0.260	−0.023	0.072	−0.154
Clear communication	-	-	-	-	-	-	1.000	0.084	0.044	−0.029	−0.112
Well-planned intervention, flexibility	-	-	-	-	-	-	-	1.000	−0.128	0.259	−0.233
Being skilful	-	-	-	-	-	-	-	-	1.000	0.031	0.056
Evaluation, self-analysis	-	-	-	-	-	-	-	-	-	1.000	−0.094
Commitment to improvement	-	-	-	-	-	-	-	-	-	-	1.000

*Source:* Adapted from Ndlela, M., 2022, ‘Preparedness for sound clinical judgement in practice: Newly Qualified Primary Health Care Nurses’, Master’s thesis, Dept. of Nursing, University of Johannesburg, viewed 20 August 2024, from https://hdl.handle.net/10210/503861

### Noticing

With noticing, three sets of correlation analyses were done: correlation between noticing and interpreting, noticing and responding and noticing and reflecting *in* action and *on* action. The results of the correlation analysis indicated a strong correlation coefficient between focused observation and recognising deviations from the expected pattern, with rho 0.448; between focused observation and information seeking, the correlation coefficient was rho 0.030, reflecting no linear correlation between the two factors. The correlation coefficient between focused observation and prioritising data was rho 0.166, and there was thus a weak correlation between the two factors of noticing. This means NQPC nurses were able to notice changes, but few were able to interpret, and even fewer were able to respond to the changes they noticed.

### Interpreting

There was a weak correlation of rho 0.133 between prioritising data and making sense of data. The respondents could not interpret data, resulting in an inability to make sense of data. There was a further weak correlation (rho 0.112) between prioritising data and acting in a calm, confident manner. This indicated that respondents did not prioritise data as expected and could not act as required. There was a negative correlation (rho -0.013) between prioritising data and having a well-planned intervention or flexibility.

### Responding

There was a weak correlation (rho 0.260) between acting in a calm, confident manner and well-planned intervention or flexibility, indicating that acting calm has no relation to having a well-planned intervention and being flexible. The correlation analysis on acting calmly, confidently and clearly communicating was weak (rho 0.119). Respondents who acted calmly did not necessarily offer clear communication in the process.

### Reflecting on action

The correlation coefficient was weak in this area; between self-evaluation or analysis and commitment to improvement, the rho was -0.094. The results indicate that being able to self-evaluate or analyse and reflect on performance had a minimal relationship with committing to improving. Even though respondents with well-planned interventions were able to self-evaluate or analyse, there was no relationship to a commitment to improving.

## Discussion

### Noticing

The noticing dimension is important in grouping and connecting relevant data to produce constellations of individual cues, validate the diagnosis and identify potential complications (Betts et al. 2019:24). The respondents were able to make better clinical judgements in simple cases. They could notice better in a case with one medical condition; when a case had two underlying conditions, it became more difficult. The findings are consistent with other studies where advanced beginners overlooked the peculiarities of the circumstance and rendered context-free clinical judgements (Betts et al. 2019:24). Greater complexity in patients’ conditions and comorbidities pose a challenge to newly qualified nurses, as it becomes difficult to critically analyse and make sound clinical judgements (Jessee 2021:54; Van Graan et al. 2016:35). In a simple typical case, the respondents were able to notice at the exemplary level. The more complex the case became, the more difficult it was for the NQPC nurses to notice all changes as they relied on the identifiable, measurable parameters of the patient’s condition to guide their decisions. The management protocols in PHC facilities stipulate addressing problems separately, hence the unsatisfactory responses, as respondents were unable to identify which medical condition took priority over the other. Both conditions are common in PHC facilities but most often present alone. The more complex the case became, the more difficult it was for the NQPC nurses to notice all changes as they relied on the objectifiable measurable parameters of the patient’s condition to guide their decisions. Newly qualified primary care nurses should be supported at all levels in acquiring knowledge and skills. Nurse managers should allocate an experienced primary care nurse to mentor NQPC nurses so they get a chance to learn new things, practise existing skills and develop behaviour patterns and critical thinking.

### Interpreting

The NQPC nurses were able to interpret what was noticed. In Case study 2, the respondents failed to interpret what they saw accurately, and this could lead to wrong treatment plans and incorrect management. Failure to accurately interpret is made worse by a lack of knowledge, delayed and inaccurate communication (Betts et al. 2019:24). These findings are like Benner’s model and findings in other studies (Benner 1984:133; Yang et al. 2019:5).

The interpreting phase highlighted responses at the beginner level, with NQPC nurses demonstrating challenges with the complexities and multiple comorbidities presented in Case study 2. As beginners, they had insufficient and inaccurate knowledge and limited experience (Dutra & Guirardello 2021:2404; Murray, Sundin & Cope 2019:2544). In this case, the findings were consistent with other studies where respondents could notice changes in simple cases but experienced challenges in interpreting changes (Dutra & Guirardello 2021:2404; Murray et al. 2019:2544). Interpreting observed data is important in making sense of and drawing patterns to develop justified plans for intervention. The interpretation is based on knowledge and past experiences. The findings indicate that even though NQPC nurses can notice changes in the patient’s condition, they cannot prioritise the data while planning to manage the patient. The more complex the clinical condition becomes, the less likely newly qualified PHC nurses are to prioritise the management plan. These findings are like Benner’s model and other studies (Benner 1984:133; Yang et al. 2019:5). Attending regular clinical reviews will assist in managing unfolding patient conditions. Integrating physiology and pathophysiology knowledge into patient condition should also be encouraged. It reinforces knowledge of normal and abnormal clinical features and strengthens the ability to interpret while stimulating the intuition of possible changes and interventions. Interpreting should not only focus on initial changes that were noticed but also on unfolding changes (whether positive or negative) and should continue until the patient is stabilised or discharged.

### Responding

In Case study 1, most respondents were at an exemplary level as they carried out a well-planned intervention, were flexible to unfolding events and mastered the skill of managing a dehydrated child. These findings contrasted with the more complex Case study 2, where few respondents were able to carry out a well-planned intervention, and they were inflexible. A small percentage of 3.9% mastered the skill of managing at an exemplary level. All other respondents were functioning at an advanced beginner level as they were challenged by the complexities and multiple comorbidities presented in Case study 2. The respondents demonstrated insufficient and inaccurate knowledge with limited experience (Dutra & Guirardello 2021:2404; Murray et al. 2019:2544). The study’s findings are consistent with Benner’s model, which posits that newly qualified nurses have a holistic understanding of the situation at hand but lack speed and flexibility in responding. The findings are further substantiated by Jessee (2021:54), who reported that 23% of newly employed nurses were unable to demonstrate entry-level competency and failed to reach clinical judgements, which were crucial for patient care.

The findings are consistent with other studies that reported NQPC nurses have challenges applying their knowledge in the rapidly changing and complex clinical setting because they lack the situational understanding required to increase their readiness to practice (Benner 1984:4; Kinyon et al. 2021:600; Strickland et al. 2017:86). The NQPC nurses should be supported by allocating them to attend various in-house and external clinical practice courses, including continuous professional development programmes that are clinical-related and undergo routine performance management and development reviews. Nursing management should make all relevant health policies and guidelines available as a reference for all staff and NQPC nurses.

### Reflecting

Newly qualified nurses could reflect correctly on their knowledge gaps and room for improvement in Case study 1, but failed to do so in Case study 2. This is challenging as failure to reflect correctly implies that they thought they had done exceptionally well, whereas, they have failed to make sound clinical judgements. They could not use the experience they gained to build on future responses as a sign of development (Benner 1984:133). The self-reporting scores on the LCJR were higher than the actual performance scores, and this may have a negative impact as self-confidence may lead to unsafe patient care. The self-rated responses on reflection contrasted with the clinical judgements made in managing the cases, as Case study 1 was well managed while Case study 2 was poorly managed.

The descriptive analysis of both scores illustrated that only 50.2% of the NQPC nurses in public PHC clinics in the three sampled health districts of Gauteng were functioning at exemplary levels and were able to make sound clinical judgements in practice, ensuring safe patient care. Conversely, 29.4% of NQPC nurses were at the developing level, and 20.4% were at the beginner level. Nursing education should integrate instructional strategies that stimulate and strengthen cognitive thought processes among students. Various teaching strategies, including chunking and scaffolding clinical learning practice, will allow the NQPC nurses to synthesise the learning content.

### Recommendations

Nursing education should use high-fidelity simulations and unfolding case studies to stimulate critical thinking (Hensel & Billings 2020:130). Nurse educators should become critical thinkers to cultivate the virtue of critical thinking and intellectual curiosity (Dickison, Haerling & Lasater 2019:77). Nursing education should be student centred, as nurses must develop higher-order thinking skills while preparing to make sound clinical judgements (Wright & Scardaville 2021:8). Clinical policies for a mentoring and coaching programme for all NQPC nurses with a designated clinical mentor in practice to ease their transition from student to NQPC nurses are required (Jessee 2021:51; Maphumulo & Bhengu 2019:1). Integrating physiology and pathophysiology knowledge into patient condition should also be encouraged as it reinforces knowledge of normal and abnormal clinical features and strengthens the ability to interpret while stimulating the intuition of possible changes and interventions. Interpreting should not only focus on initial changes that were noticed but also on unfolding changes (whether positive or negative) and should continue until the patient is stabilised or discharged.

### Further research

A further longitudinal cross-sectional comparative study of NQPC nurses is recommended. The study should be conducted at different post-qualifying periods to identify a stage where all NQPC nurses are functioning at an exemplary level. This longitudinal study could compare the progress made by each NQPC nurse.

### Limitations

The study focused on NQPC nurses in public PHC facilities in three districts of Gauteng. The study had an unforeseen limitation as none of the facilities that were visited by the researcher had NQPC nurses who completed their training in private nursing colleges or universities. The sample was restricted to NQPC nurses who qualified within the 12 months before data collection as other studies extended the period of newly qualified to 18 months.

## Conclusion

Clinical judgement of NQPC nurses entering practice is often perceived as not meeting expectations for providing quality patient care. This study’s findings show that while NQPC nurses possess theoretical knowledge and clinical skills, they can notice salient changes in the patient’s deteriorating condition but fail to interpret the changes they have seen correctly, thus failing to respond appropriately to the patient’s needs. Failure to respond to patient’s needs leads to poor-quality care and complications. To facilitate the preparedness of NQPC nurses’ clinical judgement, programmes for clinical training must be improved by adding more practical experience and simulation-based learning, while clinic managers and mentors should conduct regular competency evaluations and facilitate attendance of in-house professional development programmes. Furthermore, NQPC nurses should be provided with designated mentors to support the development of improved clinical judgement. The preparedness of the NQPC nurses should be approached with a solution-oriented mindset of recognising the potential for improvement by introducing mentorship programmes, ongoing training initiatives and technologically structured support systems that facilitate the development of clinical judgement skills needed for practice.

## Appendix 1

### Preparedness for Sound Clinical Judgement in Practice

#### Newly Qualified Primary Health Care Nurses

**Case Study 2:** A 50-year-old diabetic patient is brought to the clinic at 07:30. The patient is unresponsive to all stimuli. There is a history of the patient having diarrhoea for one day. The patient is on oral diabetic treatment.

5.1 What additional history would you collect from the family/escort regarding the patient’s condition?
  a)
  b)
  c)
  d)
5.2 What examination would you conduct on this patient?
  a) □ Blood Glucose level (HGT)
  b) □ Blood Pressure (BP)
  c) □ Urinalysis
  d) □ Weight
5.3 What would your differential diagnosis of the patient be?
  a) □ Hypoglycaemia
  b) □ Hyperglycaemia
  c) □ Keto-acidosis
  d) □ Dehydration
5.4 What other symptoms would confirm your diagnosis of this patient?
  a)
  b)
  c)
  d)
5.5 Justify your diagnosis.
  a)
  b)
  c)
  d)
5.6 What management would you give/prescribe to the patient?
  a) □ Synthetic Insulin
  b) □ Intravenous infusion of Normal Saline with 20mls of 50% Glucose
  c) □ Oral glucose solution
  d) □ Intravenous infusion of Ringer’s lactate
  e) □ 10% Dextrose infusion
5.7 After 2 h, the patient is responsive, and the Blood Glucose reading is 4.2 mmol/L. Re-arrange and prioritise the management of this patient.
  a) □ Give oral glucose
  b) □ Give a snack
  c) □ Test Urine
  d) □ Check weight
5.8 What are the complications of this condition for the patient? List 4 only.
  a)
  b)
  c)
  d)
5.9 What further management will this patient require? List 3 only.
  a)
  b)
  c)
5.10 Justify why the patient would need management listed above.
  a)
  b)
  c)
5.11 Reflect on what else you think you could have done for this patient.
  a)
  b)
  c)
  d)

Thank you for taking the time to complete this questionnaire and for your participation in this research study. Please place in the envelope provided and seal.
